# Case of Tubercular Peritonitis Complicated by Periosteal Abscess of the Thigh—Laparotomy—Recovery

**Published:** 1894-01-06

**Authors:** A. G. Miller

**Affiliations:** Lecturer on Clinical Surgery and Surgeon to the Royal Infirmary, Edinburgh


### CASE OF TUBERCULAR PERITONITIS COMPLICATED BY PERIOSTEAL ABSCESS OF THE THIGH. — LAPAROTOMY. — RECOVERY.

By A. G. Miller, M.D., F.R.C.S.E ; Lecturer on Clinical Surgery and Surgeon to the Royal Infirmary, Edinburgh.

This case is reported as an admirable example of what is not unfrequently met with in children, viz., a rapid and complete recovery after laparotomy for tubercular peritonitis.

It is especially interesting, as just four years have elapsed since the operation was done, and the disease has not recurred.

The child was seen a few months ago, and was quite well and strong, the picture of health, fat and ruddy. Her bowels were acting regularly, and she complained of nothing whatever.

The following points in this case are worthy of notice:

1. A periosteal abscess was the occasion of her being brought to the hospital, though it was undoubtedly secondary to the peritonitis. It ran its course (a very favourable one), and seemed in no way to affect, or be affected, by the peritonitis.

2. The peritonitis was not dry but purulent, the pus being in considerable quantity. The pus was partly limited by adhesions, but collected by gravitation in considerable quantity in Douglas's pouch. It also presented anteriorly near the umbilicus, but did not collect in the flanks.

3. After free evacuation the secretion of pus almost ceased, and the cavity in which it had been contained contracted with such rapidity that in about six weeks it was practically obliterated.

4. It is worth while noting the effect of closing the wound at the operation, and the improvement in the patient's condition produced by opening up the wound and putting in a drainage tube.

5. The immediate and satisfactory progressive improvement, with complete and speedy recovery, are worth remembering. So good a result is most common in purulent cases, and those in which adhesions are not extensive, and the intestinal glands are not affected.

Notes taken from records of Wards XXXIII. and XVII.:—

Maggie F., aged ten, residing at Broxburn. Admitted to Ward XVII., Bed XIII., February 11th, 1890, complaining of a pain in her left leg. Sent by Dr. Wyllie from Ward XXXIII. Medical.

*History.*—Three weeks past last Tuesday patient had some dumpling to eat, which she was not accustomed to. Next day she had violent pains in the abdomen and vomited. The vomiting stopped on the following day, but the pain continued. During this time she also had an attack of diarrhœa. On the following Tuesday she began to complain of pain in the leg.

*Present Condition.*—Left leg flexed and slightly everted. The thigh is considerably swollen, and there is a distinct feeling of fluctuation. Patient is very emaciated, and the abdomen is tumid. There are marks of old suppurations (glandular) in the neck. There is diarrhœa.

*Treatment.*—On the 13th Mr. Miller opened the abscess at two points, passing a drainage tube from the one opening to the other and washing out the cavity.

The abscess contracted, and healed readily without any necrosis of bone occurring. The diarrhœa continued, however, and the abdomen became more distended. Examination per rectum revealed a protrusion downwards of Douglas's pouch by fluid. From these symptoms tubercular peritonitis was diagnosed, and permission asked from the patient's friends for a laparotomy.

By March 9th there was considerable redness and protrusion of the umbilicus. To avoid rupture, leave for laparotomy not having been obtained, Mr. Miller incised the swelling of the umbilicus, and evacuated a considerable quantity of pus.

On March 17th, the consent of the parents having been obtained, Mr. Miller opened the abdomen a little below the umbilicus by means of an incision about three inches in length, evacuating a large quantity of pus, and then washed out the cavity with warm boracic lotion. The intestines were much matted together, but no attempts were made to break down the adhesions. The washing out was continued till the lotion returned quite clear, and Douglas's pouch was ascertained to be empty. The wound was then stitched up and dressed.

After the operation the patient did not complain of pain, and the temperature remained satisfactory till late at night, when it suddenly went up to 102·4 degrees. Next day, on account of the temperature, Mr. Miller opened up the wound, giving vent to a considerable quantity of bloody serum, and inserted a glass tube. Three days later the tube was removed because the wound was gaping, and there was free communication with the abdominal cavity. By March 27th the wound was apparently superficial, and by April 29th it was a mere line.

After the operation the diarrhœa stopped, and the patient began to improve visibly. On June 17th, 1891, she was shown to the Edinburgh Medico-Chirurgical Society, when she was stout and well, and was attending school regularly.

